# Consistency in personality trait judgments across online chatting and offline conversation

**DOI:** 10.3389/fpsyg.2023.1077458

**Published:** 2023-05-05

**Authors:** Wenjie Wu, Peter Mitchell, Yingguo Lv

**Affiliations:** ^1^Department of Psychology, Lingnan Normal University, Zhanjiang, China; ^2^School of Social Sciences, University of Bradford, Bradford, United Kingdom

**Keywords:** personality trait judgments, online chatting, real-world conversation, judgmental consistency, person perception

## Abstract

Past research has suggested that people utilize various non-verbal cues to make personality judgments in either real-world or online environments, but little is known about the extent to which a person would be perceived consistently across realistic and virtual contexts. The present study was to investigate this issue, exploring the extent to which the same target was judged consistently in terms of empathic and big-five traits across online text-based chatting and offline conversation, and to pinpoint how the judgments occurred in the two contexts. In the formal procedure, 174 participants were asked to make trait judgments and evaluate the observable cues about the partner after chatting online and after watching the partner (who the participant did not know was the same person in the online chatting) in a real-world conversation. The results demonstrated the following: (1) Participants made consistent judgments of each trait about the same target across the online chatting and the offline conversation; (2) many cues in each context were employed to drive trait judgments, whereas few cues validly revealed the self-reported assessments of the traits. The results were discussed based on the empirical and theoretical work in person perception.

## Introduction

With the widespread application of various social media (such as Facebook, Twitter, and WeChat), social life expands from traditional face-to-face interaction to diverse virtual communications, such as online chatting and sharing social activities on networking platforms. Like in the real world, people encounter others from all walks of life in the virtual environments, forming first impressions (Weisbuch et al., 2009), presenting oneself (Lee et al., 2014), and making trait judgments (Lee et al., 2014; Wu et al., 2021) from multiple observable “digital footprints.” Past research has suggested that people form consistent personality impressions of strangers across different situations occurring in the real world (e.g., Borkenau et al., 2004; Wu et al., 2016a, 2017), but little is known about the extent to which a person would be perceived consistently across realistic and virtual contexts. What cues might be available in the processes of personality judgments happening in daily conversation and in online communication? The current study sought to provide some insight into these issues.

### Personality judgments in real-world and virtual contexts

Signals and cues of facial expressions and behavior embodied in psychological dispositions (Funder, 2012; Wu et al., 2016b) allow people to accurately judge some dimensions of the big-five traits (e.g., Carney et al., 2007; Thoresen et al., 2012; Back and Nestler, 2016) as well as the extreme levels of the empathic trait (Wu et al., 2016a) and the big-five traits (Wu et al., 2017). Similarly, “digital footprints” such as nicknames, profile images, and postings left on social media embodied in real personalities (Vazire and Gosling, 2004; Marcus et al., 2006; Back et al., 2010) enable people to infer some dimensions of the big-five traits (Tskhay and Rule, 2014; Azucar et al., 2018) and to detect those who are located at the extreme levels of the big-five trait continua (Wu et al., 2021). Based on this evidence, it seems reasonable to hypothesize a correspondence in personality trait judgments across online and offline contexts.

Indeed, Weisbuch et al. (2009) reported the very first empirical work, examining the consistency in first impressions of likeability across spontaneous behavior observed in real-world interaction and information abstracted from Facebook pages. In particular, participants' judgments of how much they liked their partner during the 5-min structured conversation were consistent with the judgments of liking the partners based on observing the details appearing on Facebook pages; participants were also asked to explain what informed their judgments. The results demonstrated that people could form a consistent first impression of likability on a given target across face-to-face interaction and the Facebook pages and revealed the valid information utilized in making these judgments (which included details from Facebook pages). Yet, this study did not reveal whether a given person would be perceived consistently by the same perceiver across the online and offline contexts. In Weisbuch et al. study, the target had a face-to-face conversation with a well-trained confederate who formed the first impression from the target's spontaneous behavior; in contrast, Facebook-based first impressions were made by 10 third-party raters according to the information posted on website pages. Given that the first impressions were made by different persons, it is unclear whether the same person would form the same impressions of a given target across the two contexts.

Though considerable research has suggested the capability of inferring some dimensions of the big-five traits in either real-world or virtual contexts, no research has been reported to explore the extent to which the same person could judge personality traits of the target/partner consistently across online and offline contexts. The insight into this issue will enrich our understanding of person perception across a wide scope of social interactions (involving daily conversation and online chatting) and provide empirical evidence for examining the theories of person perception, such as the realistic accuracy model (RAM, Funder, 2012) and the social relations lens model (Back et al., 2011).

### Cue utilization in the processes of personality judgments

How do personality trait judgments occur? According to the model of interpersonal perception (Gosling et al., 2002; Vazire and Gosling, 2004), personality manifests in observable clues associated with individual identity and spontaneous behavior (including facial expressions). For example, some of the big-five personality traits are reflected in self-reported Facebook-related behavior and observable profile information (Gosling et al., 2011). Cues of physical environments (e.g., arrangements of offices and bedrooms; Gosling et al., 2002) and voice of greetings (McAleer et al., 2014) reveal what a person is like. The RAM suggests that perceivers are usually accurate in inferring a given trait when they utilize the available cues relevant to that trait (Funder, 2012). For instance, quick gaits (Thoresen et al., 2012) and frequencies of social activities posted on Facebook (Blackwell et al., 2017) enable the perceiver to make an accurate judgment of extraversion. The social relations lens model (Back et al., 2011) also confirms that observable cues displayed in social interaction allow person perception to happen.

Utilizable cues in real-world interaction usually consist of dynamically spontaneous behavior, facial expression, verbal information, and other non-verbal cues. By contrast, available cues presented on social media are in various forms, such as text-based information, pictures, videos, and combinations of two or more types (Wu et al., 2021). Informative cues are important for personality trait judgments. Pinpointing the ways by which people utilize various cues depending on the personalities judged across the online and offline environments will provide insight into understanding the processes of trait judgments (Wu et al., 2016b).

### Current research

Empathy is composed of multiple dimensions, including state empathic concern and trait empathy (Zhao et al., 2019). The former is experienced as a momentary state elicited by various situations, while the latter pertaining to a relatively stable psychological disposition varies between people (Baron-Cohen and Wheelwright, 2004; Wu et al., 2016a). The empathic trait is referred to as the ability to understand the thinking and feelings of others and to behave appropriately (Baron-Cohen, 2012). In comparison with the big-five traits, empathic trait judgment has been paid little attention in the field of social perception. Prior research indicated that people are effective in detecting low and high levels of empathy (Wu et al., 2016a) and the big-five trait continua (Wu et al., 2017), but no evidence has been reported if the same target would be judged similarly on these traits based on the real-world situations and the online settings.

The present research thus aimed to explore this issue, investigating the extent to which the same target would be formed a similar impression in terms of the big-five traits and the empathic trait across realistic and online communications. Specifically, participants were videoed when they have a 5-min structured conversation with the female experimenter on the topic of campus life. Later, they were paired with a person of the opposite gender to participate in a 10-min online text-based chatting on the same topic. Each participant was asked to infer the empathic trait and the big-five traits of the partner either after online chatting or after viewing the partner's conversation video (where they did not know the target was the same person as the online partner). They also reported the observable cues they drew upon in forming their impression in either context. Weisbuch et al. (2009) classified behavioral cues as non-verbal expressivity (e.g., lively vocal expression, smiling, open vs. closed smile, and facial expressivity) and verbal self-disclosure (e.g., revealing emotional information about the self, talking about oneself, and disclosing more than the partner) and categorized information in Facebook in terms of cues about social expressivity, a sociable interactive style, and self-disclosure. The present research focused on cues coded in the study of Weisbuch et al. but with a few modifications as detailed in the method section.

To summarize, this research was designed to examine the consistency in personality judgments between online chatting and offline conversation and to probe how the process occurs through associating observable cues with trait judgments. Given the previous evidence in the accuracy of personality judgments in real-world and virtual contexts, along with the consistency of first impressions on likability across spontaneous behavior and website pages, we hypothesized consistency in personality judgments of some traits regardless of accuracy. In addition, based on the model of interpersonal perception (Gosling et al., 2002; Vazire and Gosling, 2004) and research in the field of trait judgments (e.g., Back et al., 2011; Funder, 2012; Blackwell et al., 2017), we anticipated some cues observed either in spontaneous behavior or in online chatting would predominate in predicting trait judgments. Considering that self-report means (measurable with the inventory) of the big-five traits are generally the same as informant-report means (Kim et al., 2019), we adopted self-report means as the benchmark of accuracy. The consistency was indexed as the correlations of personality judgments relating to the same target between real-world conversation and online chatting. The study was consented by the research ethics committee of the university.

## Method

### Participants

A total of 174 college students (87 men, *M* = 20.20 years, SD = 1.22), randomly paired with the opposite gender (87 pairs), participated in the study. The sample size was prior determined following a calculation of G^*^Power 3.1 (Faul et al., 2009), offering a medium effect size (ρ = 0.30) with 95% statistical power to detect the bivariable correlations, and the sensitivity calculation with the sample size of 174 and 85% statistical power indicated an actual effect size of 0.26. The pairs of participants were previously unacquainted and had no opportunities to meet each other during the study, other than as arranged within the study.

### Materials

#### Videos

A total of 174 video clips were developed as stimuli, in each of which the participant was having a 5-min structured conversation with the female experimenter, talking something about themselves on the topic of campus life. The videos included sound and were presented in a mean duration of 4.57 min (SD = 0.59, ranging from 2.48 to 5 min) with 1920 × 1080 pixels.

#### Empathy quotient

Participants filled in the empathy quotient (EQ) (Baron-Cohen and Wheelwright, 2004), which offers a comprehensive measurement of the trait structure of empathy. It comprises 40 items (along with 20 filter items) pertaining to a range of behaviors associated with empathizing, with an overall rating as the index of individual differences in the empathic trait. The range of the scores is 0–80. All targets completed the Chinese translated version of the EQ questionnaire (adapted from the website: https://www.autismresearchcentre.com/tests/empathy-quotient-eq-for-adults/). Cronbach's α is 0.85 in the present study.

#### The NEO Five-Factor Inventory-3

The NEO Five-Factor Inventory-3 (NEO-FFI-3) (Costa and McCrae, 2011) is used to measure the five trait dimensions of neuroticism (N), extraversion (E), openness to experience (O), agreeableness (A), and conscientiousness (C). Each of the 12 items, respectively, pertains to each of the five trait dimensions, with the response to each item on a 5-point Likert scale. The range of T scores of each trait is 25–75 (and above). Cronbach's α for the traits of N, E, O, A, and C is 0.85, 0.78, 0.61, 0.60, and 0.79, respectively, in this study.

### Procedure

#### Video capture and personality measurements

In this phase, the participant individually completed the tasks for material collected in the laboratory. After signing a written consent form, they completed the EQ questionnaire and the NEO-FFI inventory in random order through the online questionnaire system Wen Juan Xing on a laptop. We calculated participants' self-ratings of the empathic trait and the big-five traits on the inventories to measure their real personalities.

An iPhone 6 was previously prepared on a tripod, about 1.5 m away to record the participant's face and the top part of their body. Then, the participant proceeded to a 5-min structured conversation with the female experimenter who sat opposite to the participant but out of view of the camera; the conversation started with a 1-min self-introduction followed by a conversation with the experimenter on the topic of campus life (e.g., What major are you studying? What do you usually do in your spare time?). During the conversation, the participant was recorded without awareness. The cover story was they would be videoed when reading aloud a verbatim script of the screen test. After the conversation, the participant was asked to read verbatim texts of a joke in front of the camera when the experimenter ostensibly switched to “record mode.” Finally, the participant posed a neutral expression for a passport-style picture while sitting on the chair in front of a white wall. The videos of reading the joke and the pictures were not used in the present research.

All participants were fully debriefed and gave written informed consent to use the videos and pictures for research purposes. Videos were edited using the software Jian Ying. Videos of the structured conversation in a duration less than 5 min were retained, and those in a duration more than 5 min were cropped from the beginning to 5 min.

#### Personality trait judgments

After 1 month of material collection, participants were paired with opposite genders at a different laboratory without any previous opportunity to meet each other. They were told their tasks were to do online chatting with an unfamiliar college student through texting, to view a video, and to fill in some questionnaires. After signing the consent form, they began with online texting for 10 min. The duration was determined by a pilot study with 26 participants (13 male participants) in which a 5-min online text-based chatting included limited information since they needed time to get to know each other. Specifically, they chatted with the partner on the topic of campus life by messaging each other on uniquely created WeChat accounts. The participants were asked not to reveal personal information (e.g., names, majors, and departments) during the chatting. Using the new accounts instead of the real WeChat accounts of the participants ensured that personality judgments were made according to online chatting rather than archived profile information.

After the chatting, the experimenter provided the information sheet of the empathic trait and the big-five traits which defined different traits, and offered an explanation of the scales (including the score ranges) of each trait based on the inventory manuals (0–80 for empathy and 25–75 for the T scores of each big-five trait dimension). Once the participants confirmed understanding the information sheet, they proceeded to the task of making personality trait judgments about the partner. The orders of the judgments about the empathic trait and the big-five traits were counterbalanced across participants, and the orders of the big-five trait judgments followed the fixed order of N, E, O, A, and C. To avoid participants anchoring their own personality ratings on the target, we adopted the continuous scales instead of the other-report inventories as the response options, that is, the participant was required to infer each partner trait by judging the trait scores using a continuous scale ranging from the lower limit to the upper limit of the scores corresponding to the trait in the inventory (i.e., 0–80 for empathy and 25–75 for the big-five traits). In addition, the participant evaluated their confidence in each trait judgment (from 0 to 100%). After completing the task of personality judgments, the participants were asked to evaluate the observable cues about the partner, with each cue presented in terms of a question that required the participant to respond on a 7-point Likert scale (details appear in the Results in the Supplementary material).

To ensure completing the same tasks synchronously by the pair of participants and to prevent being influenced by the contents of the conversation, all participants proceeded to judge the personalities of the target in the video-based conversation after the online chatting rather than in a counterbalanced order of the two contexts. Specifically, after a short break, the participant was instructed to view the partner's video in the structured conversation with the experimenter. The participant did not know the person in the video was the partner with whom they had chatted on WeChat. After watching the video, similar to the procedure in the online chatting, the participant read the information sheet and then proceeded to judge the traits of the target together with reporting their judgmental confidence. Subsequently, the participants were asked to evaluate the observable cues about the target, with each cue presented in terms of a question that required the participant to respond on a 7-point Likert scale (details appear in the Results in the Supplementary material). No participants doubted that the partner in the online chatting was the same target in the conversation video.

## Results

### Accuracy in personality trait judgments

To examine whether participants were able to detect the target trait in each of the contexts, Pearson's correlations were conducted between the self-reported personality traits and the corresponding trait ratings in the online chatting and the conversation contexts, respectively. Table 1 shows the coefficients of the Pearson correlations, along with the corresponding partial correlations controlled for the judgmental confidences (the mean judgments of each trait and the corresponding mean judgmental confidence in each context are demonstrated in Supplementary Table S1). According to Table 1, there were only significant correlations between the self-reported target E and the E rated in the online chatting (*r* = 0.16, *p* = 0.034) and the E rated in the conversation (*r* = 0.42, *p* < 0.001), and between the self-reported target N and the N assessed in the conversation (*r* = 0.16, *p* = 0.039). The results were sustained after controlling for the judgmental confidence of each trait. Overall, participants were able to detect the trait of E either in the online chatting or the offline conversation and were somewhat accurate in judging the trait of N in the conversation; but they failed to make accurate judgments on the other traits in both contexts.

**Table 1:** Coefficients (and *p*) of Pearson's correlations between self-reported personality traits and the judgments of each trait in each context, and the corresponding partial correlations controlled for the judgmental confidences, along with the 95% confidence interval of each correlation (*N* = 174).

**Traits**	**Pearson correlations**		**Partial correlations**	
	**Online chatting**	**Conversation**	**Online chatting**	**Conversation**
Empathy	0.14 (0.074) [0, 0.26]	−0.03 (0.736) [−0.17, 0.10]	0.14 (0.070) [0, 0.27]	−0.01 (0.932) [−0.15, 0.14]
N	0.01 (0.884) [−0.014, 0.17]	0.16 (**0.039**) [0.01, 0.32]	0.01 (0.889) [−0.14, 0.16]	0.16 (**0.038**) [0.0.31]
E	0.16 (**0.034**) [0.01, 0.31]	0.42 (** < 0.001**) [0.29, 0.53]	0.17 (**0.024**) [0.02, 0.31]	0.43 (** < 0.001**) [0.32, 0.54]
O	−0.10 (0.177) [−0.24, 0.04]	0.05 (0.527) [−0.10, 0.21]	−0.14 (0.075) [−0.29, 0.01]	0.06 (0.474) [−0.12, 0.21]
A	−0.01 (0.899) [−0.15, 0.13]	0 (0.971) [−0.15, 0.15]	−0.05 (0.479) [−0.20, 0.10]	0 (0.967) [−0.16, 0.15]
C	0.14 (0.063) [0, 0.27]	0.10 (0.214) [−0.04, 0.23]	0.13 (0.084) [−0.02, 0.26]	0.10 (0.186) [**-**0.05, 0.24]

The bold values correspond to the significant *p* values.

### Consistency in personality trait judgments across the online chatting and the offline conversation

Pearson's correlations for each trait judgment were carried out to examine the consistency between the online chatting and the offline conversation. The results suggested consistent judgments on each trait across the two contexts [Empathy: *r* = 0.29, *ICs* = (0.13, 0.43), *p* < 0.001; N: *r* = 0.33, *ICs* = (0.19, 0.47), *p* < 0.001; E: *r* = 0.25, *ICs* = (0.12, 0.40), *p* = 0.001; O: *r* = 0.23, *ICs* = (0.10, 0.37), *p* = 0.002; A: *r* = 0.28, *ICs* = (0.11, 0.42), *p* < 0.001; C: *r* = 0.30, *ICs* = (0.12, 0.47), *p* < 0.001]. When the self-reported trait ratings were partialed out, the correlations survived [Empathy: *r* = 0.29, *ICs* = (0.13, 0.45), *p* < 0.001; N: *r* = 0.34, *ICs* = (0.19, 0.47), *p* < 0.001; E: *r* = 0.21, *ICs* = (0.05, 0.36), *p* = 0.006; O: *r* = 0.24, *ICs* = (0.10, 0.37), *p* = 0.002; A: *r* = 0.28, *ICs* = (0.11, 0.42), *p* < 0.001; C: *r* = 0.29, *ICs* = (0.10, 0.46), *p* < 0.001]. In short, the target was estimated similarly on their traits regardless of accuracy across the online chatting and the realistic conversation.

### Cue utilization and cue validity in personality trait judgments

We examined the cues utilized when participants made judgments of each trait by calculating the correlations between the mean estimation of each trait and the mean rating of each cue and examined the valid cues by computing the correlations between the mean self-reported rating of each trait and the mean ratings of each cue (all data were transformed into Fisher's *Z* scores before calculating the correlations). Tables 2, 3 report the cue utilization and cue validity in the online chatting and the offline conversation contexts, respectively (the mean evaluations of each cue in each context are presented in Supplementary Table 2). As demonstrated in Tables 2, 3 (“cue utilization”), participants employed various cues while making trait judgments either in the online chatting or in the offline conversion, and they generally utilized more cues in the conversation than in the online chatting. For example, though few cues were utilized to judge trait N in both contexts, there were much more cues used in judging traits A and C in the realistic conversation than in the online chatting. In addition, considerable cues were adopted when judging the trait E in either context, and which cues were employed more or less depending on which of the traits was judged.

**Table 2:** Coefficients of Pearson's correlations between the mean judgments of each trait and the mean rating of each cue (cue utilization) and between the mean self-reported rating of each trait and the mean rating of each cue (cue validity) in the online chatting context (*N* = 174).

**Cue utilization**						**Cues**	**Cue validity**					
**Empathy**	**N**	**E**	**O**	**A**	**C**		**Empathy**	**N**	**E**	**O**	**A**	**C**
0.25^***^	0.06	0.46^***^	0.34^***^	0.18^*^	0.11	Contents	−0.06	−0.05	0.12	0.01	0.03	0.10
−0.08	0.01	−0.11	−0.16^*^	0	−0.07	Self-disclosure	−0.04	−0.07	0.03	0.02	0.02	−0.03
0.12	−0.05	0.33^***^	0.21^**^	0.11	0.11	Talking about self	0.03	−0.01	0.07	0.06	−0.05	0.05
0.23^**^	0.04	0.43^***^	0.28^***^	0.23^**^	0.13	Being active	−0.03	−0.19^*^	0.24^**^	−0.05	0.12	0.19^*^
0.40^***^	0.11	0.42^***^	0.31^***^	0.14	0.19^*^	Verbal skills	−0.02	−0.06	0.09	−0.03	0.05	0.12
0.27^***^	−0.01	0.30^***^	0.20^**^	0.06	0.01	Common interests	−0.10	−0.07	0.07	−0.05	0.06	0.03
0.21^**^	0.01	0.27^***^	0.10	0.13	0.12	Immediate reply	−0.03	−0.10	0.16^*^	−0.10	0.02	0.06
0.38^***^	0.17^*^	0.40^***^	0.32^***^	0.20^**^	0.07	Interesting	−0.17^*^	−0.01	0.01	−0.02	−0.03	0.02
−0.08	0.09	−0.29^***^	−0.13	−0.11	−0.05	Waiting for responses	−0.01	0.11	−0.03	0.15^*^	0	−0.01
−0.05	0.02	−0.19^**^	−0.05	−0.15	0	Using emoticons	−0.04	0	0.02	−0.07	−0.02	0.09

* p < 0.05.

** p ≤ 0.01.

*** p ≤ 0.001.

**Table 3:** Coefficients of Pearson's correlations between the mean judgments of each trait and the mean rating of each cue (cue utilization) and between the mean self-reported rating of each trait and the mean rating of each cue (cue validity) in the offline conversation context (*N* = 174).

**Cue utilization**						**Cues**	**Cue validity**					
**Empathy**	**N**	**E**	**O**	**A**	**C**		**Empathy**	**N**	**E**	**O**	**A**	**C**
0.36^***^	0.06	0.43^***^	0.40^***^	0.37^***^	0.34^***^	Contents	−0.03	−0.15^*^	0.26^**^	0.02	0.03	0.14
0.43^***^	0.04	0.52^***^	0.46^***^	0.26^***^	0.35^***^	Being active	−0.10	−0.19^*^	0.32^***^	0.05	0.08	0.22^**^
0.33^***^	0.05	0.38^***^	0.38^***^	0.29^***^	0.38^***^	Verbal skills	−0.20^**^	−0.15^*^	0.18^*^	0.05	0	0.08
0.31^***^	−0.07	0.43^***^	0.40^***^	0.26^***^	0.27^***^	Facial expression	−0.14	−0.26^***^	0.27^***^	0.10	0.08	0.20^**^
0.17^*^	0.01	0.17^*^	0.23^**^	0.33^***^	0.22^**^	Gaze attention	0.02	−0.02	0.09	−0.05	0.03	0.12
0.12	0	0.16^*^	0.20^**^	−0.01	0.16^*^	Bodily movements	0.07	0.04	0.13	−0.03	0.02	−0.05
0.12	−0.03	0.28^***^	0.23^**^	0.11	0.11	Smiling/laughing	−0.18^*^	−0.08	0.20^**^	0.04	−0.01	0.04
0.21^**^	0.085	0.35^***^	0.34^***^	0.20^**^	0.31^***^	Speaking speed	−0.19^*^	0	0.11	−0.02	−0.08	0
0.29^***^	−0.07	0.48^***^	0.44^***^	0.22^**^	0.38^***^	Confidence in talking	−0.02	−0.21^**^	0.34^***^	0.02	0.06	0.23^**^
0.34^***^	−0.03	0.39^***^	0.37^***^	0.28^***^	0.30^***^	Pleasure in talking	−0.11	−0.14	0.26^***^	−0.02	0.11	0.09
0	0.03	−0.08	−0.12	−0.08	−0.04	Self-disclosure	0.03	−0.11	−0.01	0.08	0.06	0.08
0.14	0.03	0.11	0.06	0.06	0.04	Talking about self	−0.16^*^	0.02	−0.06	−0.01	−0.15^*^	0.01

^*^p < 0.05.

^**^p ≤ 0.01.

^***^p ≤ 0.001.

By contrast, there were few cues valid in the online chatting context and limited cues valid in the offline conversation (seeing “cue validity” shown in Tables 2, 3). Specifically, in the online chatting, the self-reported N was merely negatively associated with the mean frequencies of being active, and the self-reported E was correlated only with the cues of being active and immediate replay. In the conversation context, the self-reported empathy, N, E, and C were, respectively, correlated with several cues, with more cues in E.

To probe which of the cues predicted trait judgments in each context, we conducted a linear regression analysis for each trait (except for N) and the significantly associated cues, with the trait judgments as the dependent variable and the cues as the independent variables. Table 4 displays the results demonstrating significant predictive cues. In the online chatting, the cues of verbal skills and being interesting predicted estimations of empathic traits; the frequencies of using emoticons served as the predictors in the judgments of E. There were no specific cues predicting the judgments of O, A, and C. In the offline conversation, being active was predictive for judgments of empathic trait; being active and speaking speed functioned to predict the judgments of E; speaking speed and frequencies of bodily movements predicted the judgments of O; valences of conversation contents and frequencies of gaze attention were predictive for the inferences of A, and verbal skills predicted the judgments of C.

**Table 4:** Summaries of the liner regression analyses between each trait and the corresponding correlated cues.

**IV**	**Unstandardized *B***	**SE**	***p*-value**	**95% CIs of *B***	**β**
**Online chatting**					
DV: Empathy estimations, final model: Adjusted *R*^2^ = 0.18, SE of the estimate = 11.44, *p* <0.001, *N* = 174					
Constant	25.93	5.20	**<0.001**	[15.66, 36.21]	–
Verbal skills	3.19	1.06	**0.003**	[1.10, 5.27]	0.27
Interesting	2.15	0.79	**0.038**	[0.10, 3.42]	0.19
DV: Estimations of E, final model: Adjusted *R*^2^ = 0.31, SE of the estimate = 9.79, *p* <0.001, *N* = 174					
Constant	30.38	5.78	**<0.001**	[18.96, 41.79]	–
using emoticons	−0.92	0.47	**0.049**	[−1.84, 0]	−0.13
**Offline conversation**					
DV: Empathy estimations, final model: Adjusted *R*^2^ = 0.18, SE of the estimate = 11.97, *p* <0.001, *N* = 174					
Constant	23.24	5.94	**<0.001**	[11.51, 34.97]	–
Being active	2.67	0.94	**0.005**	[0.81, 4.52]	0.30
DV: Estimations of E, final model: Adjusted *R*^2^ = 0.32, SE of the estimate =10.29, *p* <0.001, *N* = 174					
Constant	18,76	5.29	**0.001**	[8.30, 29.21]	–
Being active	2.35	0.81	**0.004**	[0.75, 3.94]	0.28
Speaking speed	2.36	1.06	**0.028**	[0.26, 4.45]	0.17
DV: Estimations of O, final model: Adjusted *R*^2^ = 0.27, SE of the estimate = 8.67, *p* <0.001, *N* = 174					
Constant	23.92	4.46	**<0.001**	[15.12, 32.72]	–
Speaking speed	1.89	0.89	**0.035**	[0.13, 3.66]	0.16
Frequencies of bodily movements	0.83	0.40	**0.038**	[0.04, 1.61]	0.15
DV: Estimations of A, final model: Adjusted *R*^2^ = 0.17, SE of the estimate = 7.56, *p* <0.001, *N* = 174					
Constant	37.99	3.75	**<0.001**	[30.58, 45.40]	–
Content valences	2.44	0.80	**0.003**	[0.87, 4.01]	0.33
Gaze attention	1.06	0.41	**0.011**	[0.25, 1.86]	0.20
DV: Estimations of C, final model: Adjusted *R*^2^ = 0.20, SE of the estimate = 8.98, *p* <0.001, *N* = 174					
Constant	29.42	4.53	**<0.001**	[20.46, 38.37]	–
Verbal skills	2.07	0.80	**0.010**	[0.50, 3.64]	0.25

The bold values correspond to the significant *p* values.

## Discussion

Past research provides considerable evidence for accuracy in judging strangers' personality traits in either realistic contexts (e.g., Back and Nestler, 2016) or in virtual environments (e.g., Azucar et al., 2018). The present research originally explored the consistency in personality trait judgments across realistic and virtual contexts, contributing to the literature by adding evidence in our ability to perceive the same person consistently in the aspects of empathy and the big-five traits across online and offline social interactions. Moreover, the study demonstrated the processes by which people utilized various cues while making personality trait judgments in realistic and virtual contexts, which enriches the theories of social perception, such as the realistic accuracy model (Funder, 2012) and the social relations lens model (Back et al., 2011). These theories are insightful in explaining the ways by which people perceive personality traits of each other by using observable cues in face-to-face interactions, whereas the current study extends the interpretive robustness into trait judgments that occur in online settings.

### Accuracy and cue validity in personality trait judgments across online chatting and offline conversation

This research is the first empirical work examining the consistency in personality trait inferences across realistic conversation and online text-based chatting (through social media WeChat), and probing the process of trait inferences on the basis of observable cues in the two contexts. The findings replicate previous reports on personality judgments in either the real world (e.g., Carney et al., 2007; Thoresen et al., 2012; Back and Nestler, 2016) or online contexts (e.g., Markey and Wells, 2002; Marshall et al., 2015; Blackwell et al., 2017), suggesting that people were accurate in detecting the trait of E. The trait E is revealed in behavioral cues. For example, extroverted people are inclined to speak loudly, which demonstrates social skills and more bodily expressions (Funder and Sneed, 1993); they also publish more social activities on social media (e.g., Blackwell et al., 2017).

According to the RAM, accuracy in trait judgments usually occurs when cues relevant to the trait are available (Funder, 2012). Our data suggested several valid cues reflecting target self-reported trait E, such as the cues of being active and frequencies of immediate reply in the online chatting, and the valence of talking contents, facial expression, being active, verbal skills, and so on in the context of the conversation (seeing Tables 2, 3). These cues were also pertaining to participants' judgments of the trait E (regardless of accuracy), some of which served to predict the judgments of E in the two contexts. In line with the RAM, when cues relevant and available to a certain trait are appropriately utilized, people are usually able to make accurate judgments on that trait.

Though the “inner trait” N is usually unperceivable from the observable cues (Vazire, 2010; Funder, 2012), participants in this study were accurate in judging N when observing the target in the offline conversation. Several cues, such as being active, the valences of talking contents, and facial expression, were negatively associated with the self-reported rating of N; however, participants seemed not to utilize the given cues to make judgments of N. Hence, the study might not capture the valid cues participants might have employed to make an accurate judgment about N in the conversation.

Despite various cues utilized in judging the other big-five traits in both contexts, there were very limited cues valid. For instance, no cues for the self-reported assessment of O were available in the conversation, and none of the cues for the self-reported assessment of A were observed in the online chatting. Although there were some cues pertaining to the self-reported C in the conversation, they did not include the cues of verbal skills which predicted the judgments of C. According to the RAM (Funder, 2012), it seems reasonable that participants found it hard to accurately judge these traits due to a lack of valid cues. Interestingly, though the cues including verbal skills, speaking speed, frequencies of smiling/laughing, and frequencies of talking about self were negatively associated with the self-reported empathic traits, these cues (except for frequencies of smiling/laughing and talking about self) were positively correlated with the judgments of empathy. According to the RAM (Funder, 2012), the cues might not be interpreted in an appropriate way; as a result, they were not informative in making an accurate judgment of empathy.

### Consistency and cue utilization in personality trait judgments across online chatting and offline conversation

Consistency was defined as the correlations in personality judgments about the same target between the online chatting and the offline conversation. There were significant consistencies in the judgments about empathic and big-five traits across the two contexts regardless of judgmental accuracy. In other words, if a person was adjudged to be an extrovert in WeChat chatting, then it was likely the person would also be adjudged extrovert in the offline conversation. Past research suggests people make use of behavioral cues in real-world situations (e.g., Borkenau et al., 2004, 2009) or “digital footprints” in virtual environments (Azucar et al., 2018) judging the big-five traits. Weisbuch et al. (2009) originally bridged the real-world context with the online environment to investigate the consistency in first impression formation, revealing that a person who was liked in a real-world conversation was also liked on Facebook. The current study extends the evidence of consistency in trait judgments about the same person across traditional conversation and text-based online chatting in terms of empathic and big-five traits. It provided a creative methodology for exploring consistent first impressions in personality traits in an extensive scope of contexts. Nowadays, people interact with each other not only in the traditional face-to-face ways but also through various social media, and thus, it is meaningful to know the extent to which we can make similar trait judgments about the same person across real and virtual environments, and how these judgments happen. The present study contributed to the insights into these issues.

Why did people make consistent trait judgments about the same person across real and virtual contexts? On the one hand, personality traits, as a stable psychological structure, are relatively consistent over time and across situations (Funder, 2006). Actual personalities can be leaked out in spontaneous behavior (e.g., Carney et al., 2007; Funder, 2012; Biesanz, 2014; Wu et al., 2016a,b, 2017) and can be traced from a variety of online information (Vazire and Gosling, 2004; Back et al., 2010; Wu et al., 2021), such as status updates (e.g., Gosling et al., 2011), online social activities postings (Blackwell et al., 2017), and profile pictures (Liu et al., 2016). The same target would more or less anchor his/her actual personality in multiple cues when having a conversation with the experimenter or when doing a text-based online chatting with the partner. On the other hand, research has revealed that people behave consistently across different social situations (Funder and Colvin, 1991). In the present research, participants utilized many cues when making judgments of each trait (except for N) in both online chatting and offline conversation. Some cues were predictive in judging a particular trait, that is, the utilization of the observable cues in both contexts enabled consistent judgments of the same trait about the same person.

The present research directly compared cues observed from the same person in both online chatting and offline conversation contexts when making personality trait judgments. These not only provide evidence for the correspondence between personality trait judgments of the same person across the two environments but also probe the processes by which participants made use of multiple observable cues to infer each of the traits.

Different from the research from Weisbuch et al. (2009) which compared spontaneous behavior with the information abstracted from the website pages, the present research constrained the contexts to social interaction, occurring either in the real world or on social media. Ongoing behavior (e.g., facial expressions, verbal contents, and bodily movements) in traditional conversation is dynamic and fleeting (Wu et al., 2021), whereas cues in text-based online chatting are presented in static patterns (such as texts and emoticons) that can be repeatedly reviewed. The data showed cues from the real-world conversation were much richer than those from online chatting. Even so, participants were able to make consistent trait judgments across the two contexts, suggesting that different types of cues were observed and employed when making judgments of personality traits. Nonetheless, participants were generally more confident in their trait judgments when observing the target in the conversation than when chatting with them online (relevant data analyses appeared in the Supplementary material).

Participants made use of various cues in judging the personality traits of strangers no matter whether the judgments were accurate. In other words, people might be driven to explain observable information and the behavior of others for understanding others' psychological dispositions (Wu et al., 2019). They base their personality trait judgments on something available about the target, which makes them feel reasonable about their behavior (embodied in personality judgments). Nonetheless, how the cues are utilized during personality judgments depends on different traits. Participants did not indiscriminately draw upon all available cues to judge each trait in the online chatting or the conversation. For instance, in the offline conversation, the cue of being active in the talking predicted participant judgments about the empathic trait, whereas the cues of verbal skills predicted the judgments of trait C.

### Limitations and future research

Given that the participants judged each other on the personality traits during the dyadic online interaction or based on viewing the partner in a dyadic conversation, the research involves interdependent data that might be influenced by the potential covariance (Gonzalez and Griffin, 2012), such as following the general opinion about others when ascribing personality traits to each other. This possibility cannot be completely excluded, though we did the partial correlations by controlling self-reported assessments of each trait when calculating the consistencies of each trait rating across the online and offline settings. Hence, future research can adjust the experimental design by asking each participant to, respectively, interact with two partners in the online setting and then meet and interact with one of the online chatting partners and with a new partner in real-life contexts. In this way, it would provide a direct examination of the difference with which the participants evaluate the same person compared to a person who actually has different characteristics. If the high correlation is only observed when the partner was the same rather than when the partner is a different person, then the consistency in the present research would be robust and corroborated.

## Conclusion

Despite that people were not good at inferring empathic traits and most dimensions of the big-five traits while having a brief texted-based online chat or observing the partner in a real-world conversation for several minutes, they formed pretty consistent first impressions of empathy and the big-five traits on each other across the virtual and realistic social contexts. Few valid cues were associated with targets' real personality traits, which makes it difficult to accurately judge most traits except for the trait E which was signaled in several cues in online and offline communications. Nevertheless, people did make use of quite a few cues while judging the partner during online chatting or viewing the partner in the face-to-face conversation, and which cues were utilized depending on which trait was judged. Furthermore, more cues were employed in the offline conversation than in the online chatting while making judgments of each trait, and some cues served to predict the judgments of some traits. In conclusion, the present research provided an original empirical examination of consistency in trait judgments across online chatting and offline conversation and illuminated the process by which people made use of various cues in judging each of the traits. The research adds insight into social perception in real-world and virtual social interactions, reflecting our capability in perceiving others (even strangers) consistently.

## Data availability statement

The datasets presented in this study can be found in online repositories. The names of the repository/repositories and accession number(s) can be found at: https://doi.org/10.6084/m9.figshare.22321432.

## Ethics statement

The studies involving human participants were reviewed and approved by the Ethics Committee of Lingnan Normal University. The patients/participants provided their written informed consent to participate in this study.

## Author contributions

WW contributed to the conceptualization, methodology, data analyses, writing, and reviewing the original draft. PM contributed to reviewing the draft. YL contributed to data collection and data analyses. All authors contributed to the article and approved the submitted version.
