# Supplementary material for: Consistency in personality trait judgments across online chatting and offline conversation
Source: Front Psychol. 2023 May 5;14:1077458. doi: 10.3389/fpsyg.2023.1077458 (PMC10195996; doi:10.3389/fpsyg.2023.1077458)
Supplement: Supplementary file 1 [file Data_Sheet_1.docx]

**Supplementary Material**

**Method**

**Ratings of cues in online (WeChat) chatting on 7-point scales**

1. Valences of contents: How positive were the chatting contents provided by the partner? (from “extremely negative” to “extremely positive”)
2. Self-disclosure: To what extent the partner would like to disclose real thoughts and feelings? (from “very unwilling” to “very willing”)
3. Frequencies of talking about self: How often did the partner talk about the self? (from “very few” to “very often”)
4. Being active: How active was the partner in the chatting? (from “very inactive” to “very active”)
5. Verbal skills: How were the partner’s verbal skills? (from “very bad” to “very good”)
6. Having something in common: How many commons (including hobbies, thoughts and feelings) did the partner share with you? (from “very few” to “a lot”)
7. Immediate reply: How quickly did the partner reply your messages? (from “very slow” to “very fast”)
8. Being interesting: How interesting were the chatting contents provided by the partners? (from “very boring” to “very interesting”)
9. Frequencies of waiting for responses: How often did you wait for the partner’s response? (from “very few” to “very often”)
10. Frequencies of using emoticons: How often did the partner use emoticons during the chatting? (from “very few” to “very often”)

**Ratings of cues in offline conversation on 7-point scales**

1. Content valences: How positive were the conversational contents provided by the person (in the video)? (from “extremely negative” to “extremely positive”)
2. Being active: How active was the person in the conversation? (from “very inactive” to “very active”)
3. Verbal skills: How were the person’s verbal skills? (from “very poor” to “very good”)
4. Facial expression valences: How was the facial expression of the person? (from “extremely negative” to “extremely positive”)
5. Frequencies of gaze attention: How often did the person pay gaze attention during the conversation? (from “very few” to “very often”)
6. Frequencies of bodily movements: How often were bodily movements of the person? (from “very few” to “very often”)
7. Frequencies of smiling/laughing: How often did the person smile/laugh? (from “very few” to “very often”)
8. Speaking speed: How was the speaking speed of the person? (from “very slow” to “very fast”)
9. Confidence of talking: How confident in the conversation was the person? (from “very unconfident” to “very confident”)
10. Pleasure of talking: How pleasure was the person in the conversation? (from “very unpleasure” to “very pleasure”)
11. Self-disclosure: To what extent the person would like to disclose real thoughts and feelings? (from “very unwilling to “very willing”)
12. Frequencies of talking about self: How often did the person talk about the self? (from “very few” to “very often”)

**Results**

Table S1 Mean trait judgments (and standard deviation) and corresponding judgmental confidence of each personality trait in the online chatting and the offline conversation respectively, and the mean self-report ratings of each trait, along with the corresponding 95% Confidence Intervals (*N* = 174)

| Traits | Contexts | | | | |
| --- | --- | --- | --- | --- | --- |
|  |  | Online Chatting | | Offline Conversation | |
|  | Self-reported Trait scores  *M*（*SD*）*CIs* | Trait Judgments  *M*（*SD*）*CIs* | Judgmental Confidence  *M*（*SD*）*CIs* | Trait Judgments  *M*（*SD*）*CIs* | Judgmental Confidence  *M*（*SD*）*CIs* |
| Empathy | 40.85（9.57）  [39.42, 42.28] | 52.70（12.61）  [50.81, 54.59] | 68.69（15.96）  [66.30, 71.08] | 51.63（13.24）  [49.65, 53.61] | 69.64（15.37）  [67.34, 71.94] |
| N | 56.05（10.58）  [54.46, 57.63] | 51.45（11.11）  [49.79, 53.12] | 66.73（16.12）  [64.32, 69.14] | 51.97（11.39）  [50.27, 53.68] | 71.89（13.48）  [69.87, 73.91] |
| E | 50.04（11.60）  [48.30, 51.78] | 56.05（11.81）  [54.28, 57.82] | 71.79（15.13）  [69.53, 74.04] | 53.81（12.44）  [51.95, 55.67] | 75.74（12.62）  [73.85, 77.67] |
| O | 52.78（9.27）  [51.39, 54.17] | 54.09（10.02）  [52.59, 55.58] | 67.55（15.52）  [65.22, 69.87] | 54.29（10.13）  [52.77, 55.80] | 70.67（13.39）  [68.67, 72.68] |
| A | 46.64（9.43）  [45.23, 48.05] | 59.56（9.10）  [58.20, 60.92] | 69.81（15.95）  [67.42,72.20] | 59.19（8.29）  [57.95, 60.43] | 71.95（13.53）  [69.92, 73.97] |
| C | 42.44（10.40）  [40.89, 44.00] | 56.83（10.24）  [55.30, 58.36] | 66.74（18.13）  [64.03, 69.45] | 57.93（10.06）  [56.42, 59.43] | 71.47（15.09）  [69.21, 73.73] |

**Analyses of judgmental confidence**

To examine participant judged the traits of others more confidently in the realistic environment than in the online environment, paired-sample *t* tests were conducted in the judgmental confidence of each trait across the two contexts. Results indicated that participants were more confident in judgment most of the traits in the conversation context than in the online chatting context (Empathy: *t* (173) = 0.80, *p* = 0.422; N: *t* (173) = 4.39, *p* < 0.001, *dz* = 0.33; E: *t* (173) = 3.55, *p* < 0.001, *dz* = 0.27; O: *t* (173) = 2.68, *p* = 0.008, *dz* = 0.20; A: *t* (173) = 1.90, *p* = 0.059; C: *t* (173) = 3.52, *p* = 0.001, *dz* = 0.27).

Table S2 Mean (and *SD*) ratings of each cue (on a 7-poing scale) with the corresponding 95% Confidence Intervals (*CIs*) in the online chatting and the offline conversation contexts (*N* = 174)

| Online Chatting | | Offline Conversation | |
| --- | --- | --- | --- |
| Cues | *M* (*SD*) *CIs* | Cues | *M* (*SD*) *CIs* |
| Content Valences | 5.53 (1.11)  [5.36, 5.70] | Content Valences | 5.34 (1.11)  [5.17, 5.51] |
| Self-disclosure | 4.12 (1.69)  [3.87, 4.37] | Being active | 4.33 (1.82)  [4.10, 4.55] |
| Frequencies of talking about self | 4.48 (1.60)  [4.24, 4.72] | Verbal skills | 5.08 (1.19)  [4.90, 5.26] |
| Being active | 4.98 (1.63)  [4.73, 5.22] | Facial expression valences | 5.02 (1.26)  [4.83, 5.21] |
| Verbal skills | 5.18 (1.08)  [5.02, 5.34] | Frequencies of gaze attention | 4.74 (1.61)  [4.50, 4.98] |
| Having something in common | 4.17 (1.24)  [3.98, 4.35] | Frequencies of bodily movements | 4.08 (1.80)  [3.81, 4.35] |
| Immediate reply | 5.10 (1.52)  [4.87, 5.32] | Frequencies of smiling/laughing | 4.23 (1.87)  [3.95, 4.51] |
| Being interesting | 4.26 (1.38)  [4.06, 4.47] | Speaking speed | 3.98 (0.87)  [3.85, 4.11] |
| Frequencies of waiting for responses | 3.64 (1.79)  [3.37, 3.91] | Confidence of talking | 4.64 (1.36)  [4.44, 4.85] |
| Frequencies of using emoticons | 2.52 (1.71)  [2.26, 2.77] | Pleasure of talking | 4.91 (1.19)  [4.73,5.09] |
|  |  | Self-disclosure | 4.35 (1.82)  [4.07, 4.62] |
|  |  | Frequencies of talking about self | 4.74 (1.40)  [4.53, 4.95] |
